# Impact of nutritional stress on the honeybee colony health

**DOI:** 10.1038/s41598-019-46453-9

**Published:** 2019-07-12

**Authors:** B. Branchiccela, L. Castelli, M. Corona, S. Díaz-Cetti, C. Invernizzi, G. Martínez de la Escalera, Y. Mendoza, E. Santos, C. Silva, P. Zunino, K. Antúnez

**Affiliations:** 1 Departamento de Microbiología, Instituto de Investigaciones Biológicas Clemente Estable, Av. Italia 3318, CP 11,600, Montevideo, Uruguay; 2 Bee Research Laboratory United Stated Department of Agriculture, United States of America, Center Road 306, CP 20,705, Beltsville, Maryland United States of America; 3 Sección Apicultura, Instituto de Investigación Agropecuaria, Route 50 km 11, CP 39173, Colonia, Uruguay; 4 Sección Etología, Instituto de Biología, Facultad de Ciencias, Iguá 4225, CP 11400, Montevideo, Uruguay

**Keywords:** Microbial ecology, Pathogens

## Abstract

Honeybees *Apis mellifera* are important pollinators of wild plants and commercial crops. For more than a decade, high percentages of honeybee colony losses have been reported worldwide. Nutritional stress due to habitat depletion, infection by different pests and pathogens and pesticide exposure has been proposed as the major causes. In this study we analyzed how nutritional stress affects colony strength and health. Two groups of colonies were set in a *Eucalyptus grandis* plantation at the beginning of the flowering period (autumn), replicating a natural scenario with a nutritionally poor food source. While both groups of colonies had access to the pollen available in this plantation, one was supplemented with a polyfloral pollen patty during the entire flowering period. In the short-term, colonies under nutritional stress (which consumed mainly *E. grandis* pollen) showed higher infection level with *Nosema* spp. and lower brood and adult bee population, compared to supplemented colonies. On the other hand, these supplemented colonies showed higher infection level with RNA viruses although infection levels were low compared to countries were viral infections have negative impacts. Nutritional stress also had long-term colony effects, because bee population did not recover in spring, as in supplemented colonies did. In conclusion, nutritional stress and *Nosema* spp. infection had a severe impact on colony strength with consequences in both short and long-term.

## Introduction

Insects play a significant role in the functioning of ecosystem processes, and pollination is one of their main ecological functions^1,2^. During recent years, pollinator decline has been reported worldwide^3,4^. In particular, honeybees (*Apis mellifera*) are among the most important insect pollinator in temperate and tropical areas, promoting the sexual reproduction of wild plants and commercial crops^5,6^. Although the number of managed colonies varies in association with different socioeconomic aspects^7–10^, high honeybee colony losses are occurring globally^9,11^. Colony losses are likely the result of the effect of multiple stressors^12,13^. However, it has been proposed that the combination of nutritional stress, infections by pathogens and pesticide exposure are among the most important driving forces^3,10,14,15^. Nutritional stress is associated with land use intensification and the expansion of monoculture agricultural areas, which deprives bees of the necessary polyfloral pollen needed to fulfill their nutritional requirements^14,16^. Pollen nutrition affects bee lifespan^17^, their immunocompetence^18^, their resistance to pathogen infection^19–22^ and behavioral transition^23,24^. Among the pathogens affecting honeybee health, *Varroa destructor*, RNA viruses^15,25,26^ and the microsporidia *Nosema ceranae*^27,28^, have the most important impact on colony losses^27,29–31^. It has been shown that colonies subjected to poor nutrition suffer from increased rates of *Nosema* spp. infection^32,33^. Those results contrasts with laboratory studies, where bees fed with pollen have higher infection levels of *Nosema* spp. although survive longer than bees fed with syrup and no protein^20,21,34^. It suggests that under laboratory conditions increased nutritional resources promote *Nosema* spp. replication, but the beneficial effects of nutrition on honeybee physiology surpass the adverse effects of the infection. These contrasting results highlight the complex relationship between the parasite and the host and the effects of the social environment of the colony.

*Eucalyptus* spp. plantations provide an ideal natural model to study the impact of nutritional stress on honeybee health since its pollen has a low crude protein percentage^33^,^35^, low lipid content^36^ and is deficient in isoleucine^35^,^37^.

We hypothesize that nutritional stress affects the health status of colonies, having consequences on colony depopulation and colony losses. In this study, we analyzed the effect of nutritional stress on colony strength and the infection level of *Nosema* spp., *V. destructor*, RNA viruses and *Lotmaria passim* during the nutritional stress period and in a long-term perspective.

## Results

### Experimental set-up

**Figure 1:**
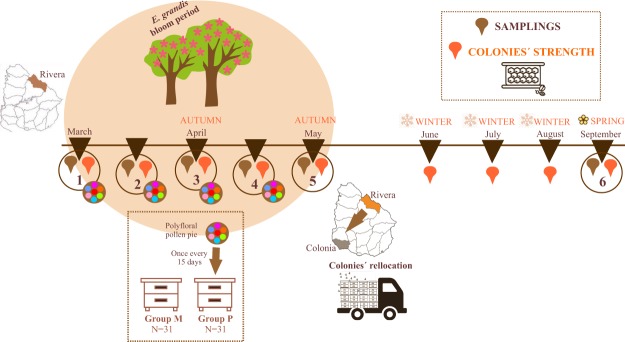
Experimental design.

To test the effects of nutritional stress on the health status of honeybees, 62 colonies were placed in a *E. grandis* plantation at the beginning of the flowering season (Fig. 1). The available pollen in the foraging area was predominantly *E. grandis*, as confirmed by the analysis of pollen collected at the hive entrance (more than 85% of pollen corresponded to these trees in all the sampling times, Supplemental Table S1). Crude protein content varied during the flowering period (26.10% in sampling 2, 17.01% in sampling 3 and 18.95% in sampling 4) and the average lipid content was 0.96%. As colonies from group M (monofloral) consumed only the pollen available in the surrounding plantation’s foraging area, they are expected to be under nutritional stress. On the other hand, colonies from group P (polyfloral) were supplemented with a polyfloral pollen patty composed of pollen from 23 different species (Supplementary Table S1), with a crude protein content of 26.31% and 3.72% of lipids. The polyfloral pollen patty showed a higher proportion of amino acids than pollen available in the environment (Supplementary Table S2).

Six pesticides were detected in the polyfloral pollen patty: azoxystrobin, atrazine, carbendazym, coumaphos, pyraclostrobin and tebuconazole. However, the concentration was closed to the detection limit of the analytic technique in all cases, and more than 208,000 times lower than LD_50_ according to the Pesticides Properties DataBase, of the University of Hertfordshire (available at https://sitem.herts.ac.uk/aeru/footprint/es/, Supplementary Table S3). On the other hand, *N. apis*, *N. ceranae*, ABPV, BQCV, DWV and SBV were not detected in the pollen patty.

### Colony pollen diversity

**Figure 2:**
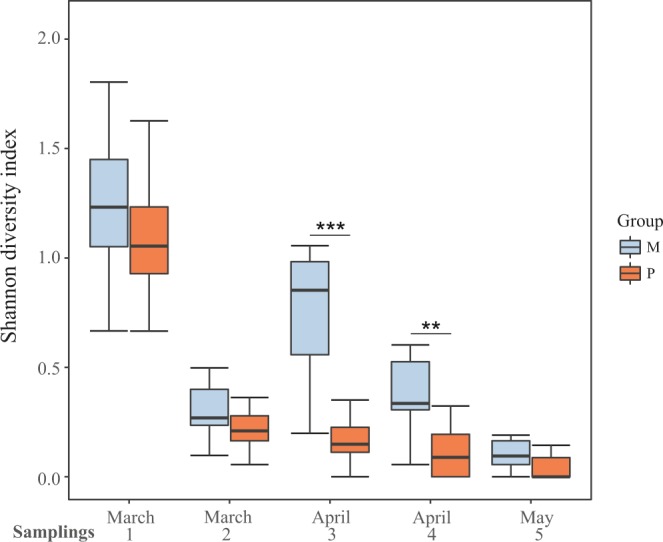
Colony pollen diversity during the nutritional stress period represented as the Shannon diversity index. Colonies from group M (monofloral) are represented in blue and colonies from group P (polyfloral) are represented in orange. Boxes show 1^st^ and 3^rd^ interquartile range and the median is represented by a line. Whiskers include the values of 90% of the samples. Significant differences of pairwise comparisons at each sampling time are represented as ** when p ≤ 0.01, and *** when p ≤ 0.001.

At the beginning of the experiment, colonies from groups P and M were collecting pollen with similar diversity (Fig. 2, Table 1). This diversity decreased significantly within 15 days in both groups (Table 1). Afterwards, colonies from group M showed higher diversity of the collected pollen than group P during the remaining flowering period, being statistically significant in sampling times 3 and 4 (Fig. 2, Table 1). The major contributor of increased forage diversity observed in group M was from two *Baccharis* spp.

**Table 1:** Statistical results of colony strength and infection level of different pathogens from groups M (monofloral) and P (polyfloral) at specific samplings (S). MW: Mann Whitney U Test. Significant results (p ≤ 0.05) are shown in black.

Comparison	Pollen diversity Shannon diversity index	Brood population	Adult population	ABPV	BQCV	DWV	SBV	*V. destructor*	*Nosema* spp. (proportion of infected bees)	*Nosema* spp. (number of spores in a pool of bees)
S1. M vs. P	MW p = 0.24	MW p = 0.57	MW p = 0.71	MW p = 0.22	MW p = 0.32	MW p = 0.23	MW p = 0.62	MW p = 0.31	T test p = 0.32	T test p = 0.94
S2. M vs. P	MW p = 0.12	**MW p = 0.05**	**MW p = 0.02**	**MW p ≤ 0.001**	MW p = 0.93	MW p = 0.81	**MW p = 0.002**	MW p = 0.5	T test p = 0.35	**T test p = 0.02**
S3. M vs. P	**MW p ≤ 0.001**	**MW p = 0.003**	MW p = 0.34	**MW p ≤ 0.001**	**MW p ≤ 0.001**	**MW p ≤ 0.001**	MW p = 0.79	MW p = 0.52	T test p = 0.12	T test p = 0.14
S4. M vs. P	**MW p = 0.002**	**MW p = 0.02**	**MW p = 004**	**MW p ≤ 0.001**	**MW p = 0.01**	**MW p ≤ 0.001**	MW p = 0.06	MW p = 0.43	**T test p = 0.004**	T test p = 0.83
S5. M vs. P	MW p = 0.06	MW p = 0.28	**MW p = 0.02**	**MW p ≤ 0.001**	MW p = 0.1	**MW p ≤ 0.001**	**MW p = 0.01**	MW p = 0.6	**T test p ≤ 0.001**	**T test p ≤ 0.001**
June: M vs. P	—	**MW p = 0.02**	**MW p = 0.01**	—	—	—	—	—	—	—
July: M vs. P	—	**MW p = 0.007**	**MW p = 0.001**	—	—	—	—	—	—	—
August: M vs. P	—	**MW p = 0.004**	**MW p = 0.02**	—	—	—	—	—	—	—
S6: M vs. P	—	MW p = 0.28	**MW p = 0.02**	MW p = 0.90	MW p = 0.41	MW p = 0.80	MW p = 0.26	—	T test p = 0.91	—

### Colony strength

**Figure 3:**
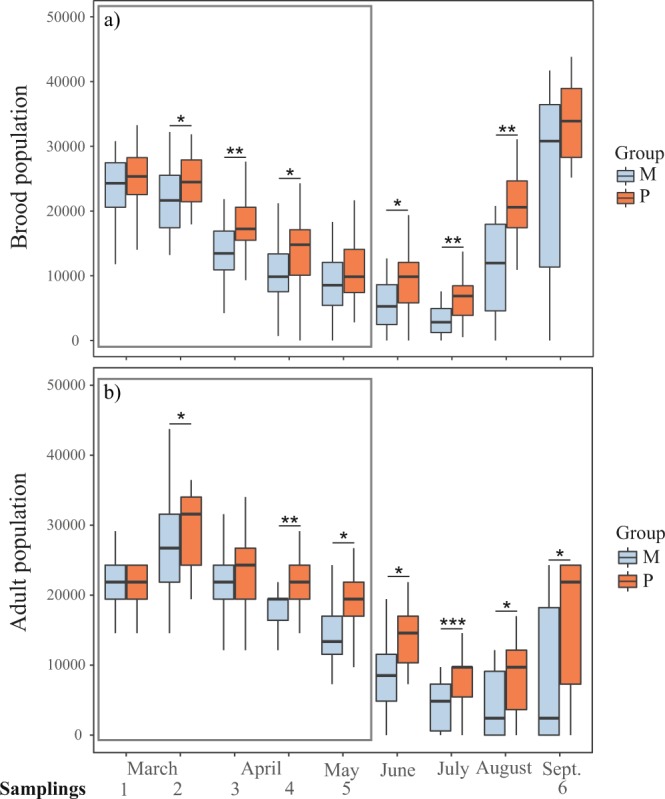
Brood (**a**) and adult (**b**) population during the nutritional stress period (squared in grey) and during the winter and spring. Colonies from group M (monofloral) are represented in blue and colonies from group P (polyfloral) are represented in orange. Boxes show 1^st^ and 3^rd^ interquartile range and median represented by a line. Whiskers include the values of 90% of the samples. Significant differences of pairwise comparisons at each sampling time are represented as * when p ≤ 0.05, ** when p ≤ 0.01, and *** when p ≤ 0.001.

Generalized linear mixed models (GLMM) were used to assess the relationship between time/treatment and colony strength/pathogen infection. Brood population was affected negatively by time, since it decreased from autumn to winter, as expected (Table 2). Treatment (pollen supplementation) affected positively brood population only in a time-manner dependence (brood = time*treatment + (1|colony) (Table 2). Pollen supplementation in autumn exerted long-term consequences since colonies from group P showed higher brood population than colonies from group M in winter (Fig. 3, Table 1). In the following spring, both groups of colonies showed similar brood populations (Fig. 3, Table 1).

**Table 2:** Statistical results of Generalized Lineal Mix Models.

Dependent variable	Independent variable	Coefficient value	Intercept value	p value
Brood population	Treatment P	0.041	10.06	0.62
	Time	−0.018		≤0.001
	Time*TreatmentP	0.005		≤0.001
Adult population	Treatment P	0.044	10.17	0.38
	Time	−0.01		≤0.001
	Time*TreatmentP	0.002		≤0.001
*Nosema* spp.	Treatment P	0.025	−0.26	0.28
	Time	0.006		≤0.001
	Time*TreatmentP	−0.001		0.02
ABPV	Treatment P	0.729	0.03	≤0.001
	Time	−0.002		≤0.001
	Time*TreatmentP	−0.0004		0.46
BQCV	Treatment P	−0.27	0.95	0.13
	Time	0,00		0.04
	Time*TreatmentP	0.002		0.34
DWV	Treatment P	0.53	0.52	≤0.001
	Time	−0.004		≤0.001
	Time*TreatmentP	0.0004		0.81
SBV	Treatment P	0.64	0.61	0.03
	Time	−0.0008		0.83
	Time*TreatmentP	0.002		0.69
Adult population	*Nosema* spp	−0.26	10.09	≤0.001
	Treatment P	0.035		0.66
	Nosema*TreatmentP	0.096		≤0.001

Sampling time and treatment also affected adult population in a similar trend as for brood population (adult = time*treatment + (1|colony). Time affected negatively the adult population (population decreased from autumn to winter), while pollen supplementation affected it positively in a time-manner dependence (Table 2).

On the other hand, there were no differences in colony mortality between both groups of colonies during the nutritional stress period (χ^2^ Test, p = 0.66). In the long-term, even though proportion of dead colonies was higher in colonies from group M (40%) compared to colonies from group P (18.5%), this difference was not statistically significant (χ^2^ Test, p = 0.12).

### *Nosema* spp. infection level

**Figure 4:**
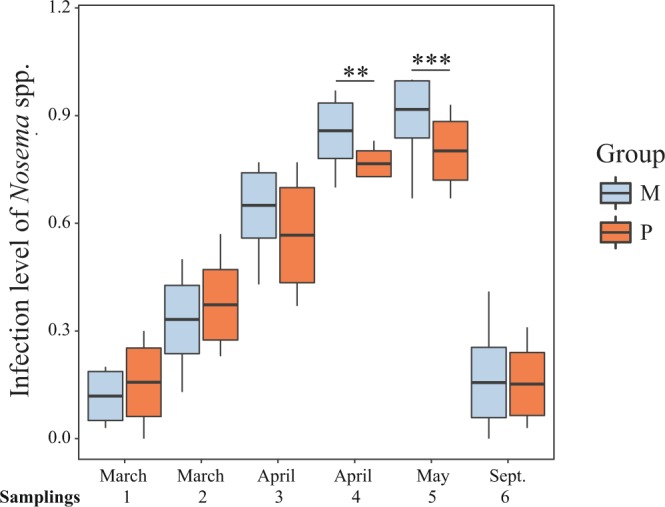
*Nosema* spp. infection level during the nutritional stress period and in spring (September). Colonies from group M (monofloral) are represented in blue and colonies from group P (polyfloral) are represented in orange. Boxes show 1^st^ and 3^rd^ interquartile range and median represented by a line. Whiskers include the values of 90% of the samples. Significant differences of pairwise comparisons at each sampling time are represented as ** when p ≤ 0.01, and *** when p ≤ 0.001.

During the *E. grandis* flowering period, pollen supplementation negatively affected the infection level of *Nosema* spp., since supplemented colonies showed lower infection level than non-supplemented colonies. This effect depends on time, since the difference in the infection level of this pathogen between both groups of colonies increased gradually throughout the sampling times (Nosema = time*treatment + (time|colony). On the other hand, *Nosema* spp. infection, increased over time (Table 2; Fig. 4).

All colonies showed similar proportion of bees infected with *Nosema* spp. at the beginning of the experiment (an average of 11.8% in colonies from group M and 15.7% in colonies from group P) (Table 1; Fig. 4). The infection increased during the *E. grandis* flowering period in both groups to a level close to 100%, while it showed the opposite pattern from sampling 5 (autumn) to 6 (spring) (Fig. 4, Table 1). When the *Nosema* spp. infection level was determined by the number of spores in a pool of 60 bees, similar results were obtained (adult = nosema*treatment + (1|colony) (Table 1).

*N. ceranae* was the most frequently detected *Nosema* species in infected colonies during the experiment. Only one colony showed co-infection with *N. apis* and *N. ceranae* in sampling 1 but in sampling 5 this colony was only infected with *N. ceranae*.

Infection level with *Nosema* spp. decreased adult population, and this effect was treatment-dependent since this effect was observed only in non-supplemented colonies (Table 2).

### Viruses and *V. destructor* infection levels

**Figure 5:**
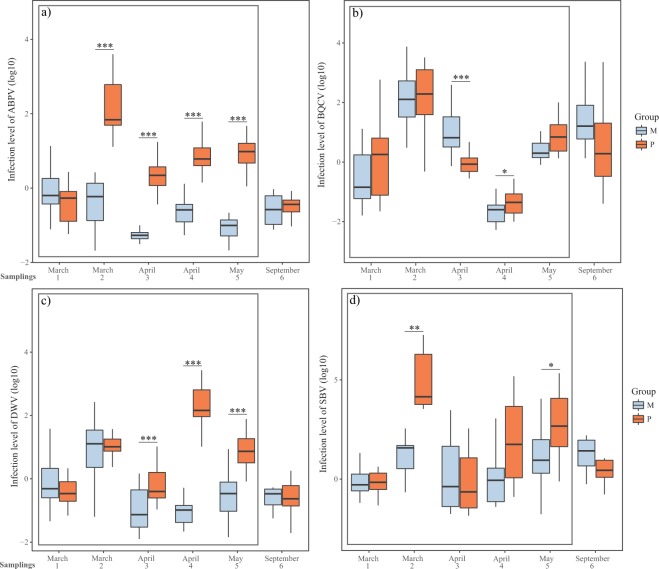
Infection level with Acute Bee Paralysis Virus (**a**), Black Queen Cell Virus (**b**), Deformed Wing Virus (**c**) and Sacbrood Bee Virus (**d**) during the nutritional stress period (squared in grey) and in spring. Colonies from group M (monofloral) are represented in blue and colonies from group P (polyfloral) are represented in orange. Boxes show 1^st^ and 3^rd^ interquartile range and median represented by a line. Whiskers include the values of 90% of the samples. Significant differences of pairwise comparisons at each sampling time are represented as * when p ≤ 0.05, ** when p ≤ 0.01, and *** when p ≤ 0.001.

The infection level with ABPV was affected by time and treatment independently since the combination of both variables did not affected it (ABPV = time*treatment + (time|colony). Pollen supplementation increased ABPV titers in all sampling times (Table 1), while time decreased it (Table 2; Fig. 5). Similar results were observed for DWV (DWV = time*treatment + (time|colony) (Table 2). Pairwise comparisons at each sampling time indicates that the treatment effect was in samplings 3, 4 and 5 (Table 1; Fig. 5). On the other hand, the infection level with SBV was affected only by treatment, with pollen supplementation having a positive effect on this variable (SBV = time*treatment + (time|colony)) (Table 2). Pairwise comparisons indicate that this effect was evident mainly in samplings 2 and 5 (Table 1; Fig. 5). Finally, the infection level of BQCV was affected only by time since the treatment effect was not consistent in the different sampling times: the infection level of this virus was lower in supplemented colonies in sampling 3 and higher in sampling 4, while there were no differences at other sampling times (BQCV = time*treatment + (1|colony)). (Table 1; Fig. 5). The nutritional stress did not have long-term consequences in virus infection levels, since both groups of colonies showed similar ABPV, BQCV, DWV and SBV infection level in spring (Table 1; Fig. 5).

*V. destructor* infestation level was similar in all colonies along the experiment and it followed its natural dynamic over time after treatment against the mite (Table 1).

### Prevalence of *L. passim*

Prevalence of *L. passim* was low at the beginning of the experiment and at the end of the nutritional stress period (lower than 10% for both groups of colonies). This prevalence increased in spring in colonies from group M (57%) while it remained low in colonies from group P (11%). The prevalence of *L. passim* was not associated with the nutritional regimen in both groups of colonies (Sampling 1 χ^2^ = 2.89 p = 0.08; Sampling 5 χ^2^ = 0 p = 0.99; Sampling 6 χ^2^ = 2.03 p = 0.15).

## Discussion

Among the multiple causes associated with the high levels of colony losses reported worldwide, nutritional stress and sanitary conditions seem to be playing a significant role in this phenomenon^3,12,14,15^. In this study, we analyzed how nutritional stress affects colony strength and health under field conditions. Colonies from group P consumed pollen available in the environment and the polyfloral pollen patty, while colonies from group M consumed only pollen available in the environment. Both pollen types were different according to their pollen diversity and nutritional properties. The polyfloral pollen patty provided bees with pollen from a diverse botanical origin, a high proportion of essential amino acids and high protein and lipid content. In contrast, the pollen available in the field was primarily composed of *E. grandis* pollen and its protein and lipid content did not satisfy the minimum requirements needed for colony maintenance and brood rearing^38^. A deficit in both protein and lipids is associated with precocious foraging^39,40^, which accelerates the aging process and leads to decreasing colony population. Pollen from *Eucalyptus* spp. is one of the species with the poorest lipid^35,41^ and has one of the highest ratios of omega 6–3 described^41,42^. Omega 3 is an essential fatty acid which deficiencies are associated with diseases and neurological disorders^43,44^. Taking these results into account, it can be considered that colonies from group M were under nutritional stress while colonies from group P had access to more abundance of pollen and also this pollen was of good nutritional value. Unexpectedly, colonies from group M collected more diverse pollen than colonies from group P. This result could be a consequence of i) a reduction in the foraging effort of the treated colonies because of the higher quantity and diversity of the pollen available they had and/or ii) an increase in the foraging behavior of colonies under nutritional stress^45,46^, which seems to be due to their capacity to sense this status and try to compensate it by collecting more pollen and from different sources.

The positive effect of pollen supplementation on the decrease of *Nosema* spp. infection in colonies located in *E. grandis* plantations has been previously reported^33^, but in this study we prove that this effect is time dependent. These results contrast with those obtained under laboratory conditions. The discrepancy between both approaches could be associated with the behavioral transition of bees in the colonies and its relationship with their nutritionally regulated physiology. In experiments performed under laboratory conditions, bees do not experience behavioral transition (because they do not have this possibility), but their physiology changes in association with nutrition^47,48^. Thus, in samplings performed in the laboratory at specific time points, bees fed with different diets are in different nutritional and physiological states. However, when bees become foragers in the field, their nutritional stores decreased and consequently their physiology changed^23,49^. In fact, Ament *et al*.^24^ proposed that most of the maturational changes in the bee physiology are associated with behavior rather than with age, and the behavioral maturation is under nutritional control^50^. Since nutrition affects the immune response^18^, it could be expected that when bees become foragers, the magnitude of their immune response is related with the food quality consumed during the nurse stage or with the minimum quantity of the food being consumed (as royal jelly) during the forager stage^51,52^. Thus, it could be expected that forager bees from supplemented colonies can mount a better immune response against *N. ceranae* than colonies under nutritional stress. This effect on the bee immunity might be triggered directly by the pollen supplementation^17,18^ or because of its contribution to the establishment of the gut microbiota, which has immunomodulation functions^53–55^. On the other hand, it should be noted that an essential mechanism for colony homeostasis is the social immune response which cannot be displayed in cage experiments^56^ and social immunity has been reported to be a defense mechanism for reducing the spread of *N. ceranae* within the colony^57^. In addition, the fact that *Nosema* spp. levels affected adult population mainly in non-supplemented colonies, suggests that the negative effects of this pathogen at colony levels are strongly associated with the nutritional status of the colonies.

Interestingly, non-supplemented colonies showed lower viral infection levels than colonies from group P. The higher infection level of viruses in supplemented colonies was not expected since it has been proposed that pollen nutrition decreased viral replication in comparison with undernourished bees^19^. However, our results agreed with Alaux *et al*.^22^ who suggested that larger physiological cell machinery might favor virus multiplication but it might also help bees to resist viral infection. Moreover, the higher levels of DWV observed in the supplemented colonies, are consistent with the lower infection levels of *Nosema* spp. also detected in these colonies, suggesting that this parasite competes for nutritional cell resources more successfully than DWV^58–60^. Thus, it could be expected that DWV could replicate better in supplemented colonies in comparison with colonies from the group M. Another possibility is that colonies from group M had higher infection level of virus than colonies from group P and these colonies were dead at the sampling time. Beyond the reason associated to the higher infection level of viruses in colonies from group P, these infections had no consequences on the colony strength parameters analyzed, since they were the group that showed higher population. This result contrast with the widely reported negative impact of viruses infections on honeybee colonies^12,15,25^. The fact that the virus levels found in this study (especially BQCV, SBV and DWV at specific sampling times) are lower than those found in the USA (Corona, pers.com.), suggest that the viral titers observed in our experiment could not have reached a minimum threshold level to decrease colony strength.

Regarding the trypanosmatid *L. passim*, it was not possible to find any association between the pathogen and colony nutritional stress. However, considering its low prevalence, this conclusion should be taken with caution and future studies should be carried out.

Nutritional stress had a long-term effect in the colonies since bee population did not recover in non-supplemented colonies as did the supplemented ones in spring. However, this long-term effect did not have consequences in honeybee health as both groups of colonies showed similar infection levels with the analyzed pathogens. Finally, since colonies were re-located in a favorable environment for bees after the nutritional stress period, it is not possible to discard that nutritional stress could have consequences on colony survival if they remained in the *E. grandis* environment.

This study shows under field conditions, how nutritional stress impacts honeybee health. Nutritional stress had a severe impact on *N. ceranae* infection. Although this infection had no consequences on colony loss during the nutritional stress period, it does affect colony strength in the short and long-term. On the other hand, the non-effect of virus infections on colonies strength in well-nourished colonies and the low level of virus detected compared to the ones found in countries from the northern hemisphere as in the USA, suggests that these infection levels are not enough to have a negative impact on colonies strength. Future studies comparing viral infection levels from countries from the south and north hemisphere should be done in order to address this hypothesis. Finally, it is important to notice that all these results were obtained in an in-depth study but performed in only one location and its repetition in multiple locations would be valuable in order to corroborate the results.

## Methodology

### Experimental design

Sixty-two colonies of honeybees *Apis mellifera* (hybrid between *Apis mellifera mellifera, Apis mellifera ligustica* and *Apis mellifera scutellata*) were placed in a single apiary in a *E. grandis* plantation (31° 15′ 58,25″S; 55° 39′ 40,32″W) in autumn, coinciding with the flowering peak of these trees (March 2015) (Fig. 1 and Supplementary Fig. S4). All the colonies had young sister queens and were standardized regarding brood population before the beginning of the experiment^61^. Colonies were treated against *V. destructor* using Amitraz (Apilab Lab, Tandil, Argentina) 45 days prior to the start of the experiment. Pollen and honey stores were removed.

Two groups of 31 colonies each (P and M) were randomly selected. In order to avoid robbing, colonies within each group were located together between them with a distance of one meter and a half in a semi circle and both groups of colonies were separated by a distance of 15 meters. Colonies from group P consumed pollen available in the surrounding plantation’s foraging area (mainly *E. grandis* pollen) and were supplemented with 500 g of a polyfloral pollen patty once every 15 days during the flowering period (2 months). Colonies from group M only consumed pollen available in the *E. grandis* plantation’s foraging area, and therefore represent colonies under “nutritional stress”.

To identify and characterize pollen available in the environment, three honeybee colonies (not belonging to the study) were positioned next to the experimental apiary with pollen traps at the entrance. Corbicula pollen from these colonies was sampled once every 15 days (coinciding with the sampling times). One portion of the pollen obtained in all sampling times was used for determination of the botanical origin, and another portion was mixed and stored at −20 °C until the nutritional pollen analysis was performed.

Polyfloral pollen patty was prepared using stored polyfloral pollen (bee bread), collected in summer 2014–2015, from healthy bee colonies belonging to the Apiculture Section of the National Institute for Agricultural Research (INIA) La Estanzuela. This pollen was homogenized in a proportion of 15:1 of pollen:sugar syrup and the mixture was divided into portions of 500 g and stored at −20 °C.

All colonies were sampled before the experiment began (Sampling 1) and once every 15 days until the end of the flowering period (Samplings 2; 3; 4 and 5). Samplings consisted of (i) forager bees stored in ethanol to detect and quantify *Nosema* spp. spores, *Nosema* species determination and *L. passim* prevalence, (ii) nurse bees to detect and quantify viral infection levels (sacrificed at −80 °C), (iii) nurses bees stored in ethanol to quantify *V. destructor* infestation level, and iv) freshly-stored pollen from the cells next to the brood area to identify colony pollen diversity. Colony strength was estimated in all colonies through the visual inspection of adult and brood population^61^.

Since colonies become notably weaker if they are not removed from *E. grandis* environments during winter^33^, all the colonies were relocated to the experimental station of INIA La Estanzuela, once the flowering period ended (34° 20′ 23.72″S–57° 41′ 39.48″W). Colonies were inspected once every month during winter and colony strength was estimated^61^. To analyze the long-term effect of nutritional stress, all colonies were sampled in spring (September, Sampling 6) as previously described. Figure 1 summarizes the experimental design.

### Pollen analysis

#### Pollen botanical origin

Samples of the polyfloral pollen patty, pollen available in the foraging area and freshly-stored pollen from the cells next to the brood area were analyzed to determine their botanical origin. At least 1200 pollen grains per sample were identified (400 grains per slide, 3 slides per sample) and the percentage of each pollen species was calculated^62^.

#### Crude protein, lipid content and amino acid composition

Samples of the polyfloral pollen patty and pollen available in the surrounding foraging area were also analyzed to evaluate their nutritional composition. For crude protein analysis, pollen samples (5 g) were dried at 60 °C, and the protein content was quantified using the Kjeldahl acid digestion technique^37^. Lipid content was analyzed as the percentage of the etheric extract^63^ and to assess amino acid composition, pollen samples were processed, derivatized and analyzed by HPLC (CBO Laboratory, Brazil)^64–66^.

#### Pesticides analysis

The polyfloral pollen patty was analyzed to detect the presence of thirty-three different pesticides including the most widely used in Uruguayan agriculture. Pollen samples (5 g) were processed for pesticide detection and quantification according to Niell *et al*.^67^.

#### Pathogens detection

Although polyfloral pollen was collected from healthy colonies, the polyfloral pollen patty was analyzed to detect for the presence of *Nosema* spp. and RNA viruses. A pollen sample (0.3 g) was processed according to the protocol described by Singh *et al*.^68^. The obtained supernatant was used for RNA extraction and viral quantification, and the pellet was used for DNA extraction and *Nosema* spp. analysis.

### Detection and quantification of *Nosema* spp. spores

The infection level of *Nosema* spp. was determined as the proportion of infected bees (N = 30) and as the number of spores in a pool of bees (N = 60) in ten randomly selected colonies per group^69^. In addition, the *Nosema* species were identified in the same colonies in samplings 1, 5 (*E. grandis* flowering period) and 6 (next spring). Forager bees (N = 20 per sample) were processed as described in Anido *et al*.^70^ and 500 µl of the homogenate was used for DNA extraction using the Purelink Genomic DNA minikit (Invitrogen, USA) according to the manufacturer’s instructions. *Nosema* species determination was carried out using the protocol described by Martín-Hernández *et al*.^71^, including negative and positive controls.

### Detection and quantification of RNA viruses

Virus detection and quantification in pollen were assessed by absolute quantification. The supernatant (900 µl) was mixed with an equal volume of chloroform and RLT buffer (Qiagen, USA) and centrifuged for 30 min at 11,000 rpm. Then, the aqueous phase was used for RNA extraction using the RNeasy mini kit (Qiagen, USA). cDNA was synthesized using 2000 ng of RNA using the High Capacity cDNA Reverse Transcription Kit (Applied Biosystems, Lithuania). For both procedures, the manufacturer’s instructions were followed. The infection levels of Acute bee paralysis virus (ABPV), Black queen cell virus (BQCV), Deformed Wing Virus (DWV) and Sacbrood bee virus (SBV) were determined by quantitative PCR (qPCR) using primers previously described^72,73^. qPCR was performed in a final volume of 10 µl containing 5 µl of the Power SYBR Green PCR Master Mix (Applied Biosystems, UK), 1 µM of each specific primer and 3 µl of cDNA. Negative controls were included as well as a standard curve which consisted of seven dilution points of a plasmid containing the amplified product. The thermal cycling program consisted of a denaturation step of 10 min at 95 °C, 40 cycles of 30 s at 95 °C and 1 min at 60 °C followed by a melting curve from 60 °C to 95 °C.

Virus detection and quantification in bees were assessed by relative quantification. Nurse bees (N = 20 per colony) were processed according to Anido *et al*.^70^, and 140 µl of the final supernatant was used for RNA extraction using the Purelink Viral RNA/DNA kit (Invitrogen, USA), following the manufacturer’s instructions. The co-purified DNA was digested with DNase I Amp Grade (Invitrogen, USA) and cDNA synthesis was performed using the High Capacity cDNA Reverse Transcription Kit (Applied Biosystems, Lithuania) according to the manufacturer’s instructions. cDNA was used to determine the infection level of ABPV, BQCV, DWV and SBV by qPCR^74,75^. qPCR was performed in a final volume of 20 µl, containing 10 µl of the Power SYBR Green PCR Master Mix (Applied Biosystems, UK), 0.3 µM of each specific primer, 2 µl of cDNA and 7.76 µl of water. Negative controls including water instead of cDNA were used in each run. An inter-run calibrator plate sample and a standard curve were also included. The geometric mean of the expression level of the housekeeping genes β-actin^76^ and RPS-5^77^ was used for the results normalization^78^. The cycling program consisted of a denaturation step of 10 min at 95 °C, 40 cycles of 15 s at 95 °C, 30 s at 50 °C (46 °C for SBV) and 30 s at 60 °C. Specificity of the reactions was checked by melting curve analysis of the amplified products (from 65 °C to 95 °C). Infection level of the different viruses were analyzed by the method described by Pfaffl^79^. All qPCR reactions were performed in a CFX96 Touch^TM^ Real-Time PCR System (Biorad).

### Quantification of *V. destructor*

*V. destructor* infestation was monitored at each sampling time with 200–300 nurse bees per colony, according to the protocol described by Dietmann *et al*.^80^.

### Detection of Lotmaria passim

DNA obtained as described previously was used to detect the presence of *L. passim* by PCR according to Arismendi *et al*.^81^. Negative and positive controls for *L. passim* were included. The prevalence of this trypanosomatid was calculated as the relation between the number of infected colonies and the total number of colonies analyzed per group and per sampling time.

### Statistical analysis

Generalized linear mixed models (GLMM) were used to assess the relationship between treatment (M or P) and time (both as fixed effects), and colony strength and pathogen infection levels (as dependent variables) (package {lme4})^82^,^83^. The identity of the colonies was considered as a random effect, and random intercept or slope was chosen in each model according to the variability of each dependent variable intra and inter colony, respectively. In the case of adult and brood population, a GLMM with poisson distribution and a log link function was used. In the case of *Nosema* spp. infection and virus titers, a GLMM with gamma distribution and a log link function were used. To assess the effect of *Nosema* spp. infection in adult population, a GLMM with poisson distribution and a log link function was done considering adult population as dependent variable and *Nosema* spp infection and treatment as independent variables.

In addition, differences in the colony strength parameters and infection levels of the different pathogens between groups P and M at different sampling times were analyzed. T-student or Mann Whitney U Test were applied if the variables fitted the assumptions of parametric statistics. Differences in the prevalence of *L. passim* between groups were analyzed using the χ^2^ Test^82^. Pollen diversity collected in the colonies was analyzed as the Shannon diversity index and differences between groups P and M and in sampling times were analyzed with Kruskal Wallis and Mann Whitney U Test (package {agricole})^82^,^84^. Differences in colony mortality between both groups of colonies were assessed using the χ^2^ Test^82^. In all cases, R Studio software was used and p-values under 0.05 were considered statistical significant.

## Supplementary information

Supplemental material

## Data Availability

The datasets generated during and/or analyzed during the current study are available from the corresponding author on reasonable request.
