# Supplementary material for: Impact of nutritional stress on the honeybee colony health
Source: Sci Rep. 2019 Jul 12;9:10156. doi: 10.1038/s41598-019-46453-9 (PMC6626013; doi:10.1038/s41598-019-46453-9)

Impact of nutritional stress on the honeybee colony health

Branchiccela B., Castelli L., Corona M., Díaz-Cetti S., Invernizzi C., Martínez de la Escalera G., Mendoza Y., Santos E., Silva C., Zunino P., Antúnez K.

Supplemental Table S1. Botanical origin of the polyfloral pollen patty and the pollen available in the *E. grandis* foraging area at the different sampling times.

		**Polyfloral pollen patty**	**Pollen available in the field** Sampling 2	**Pollen available in the field** Sampling 3	**Pollen available in the field** Sampling 4	**Pollen available in the field** Sampling 5
**Family**	**Scientific name**	**%**	**%**	**%**	**%**	**%**
Myrtacea	*Eucalyptus* spp.	3,5	91,75	87,94	86,4	99,35
Asteraceae	*Baccaris* spp. 1	0,2	1,97	1,09	0,25	-
Asteraceae	*Baccaris trimera*	-	0,12	10,6	0,15	-
Asteraceae	*Baccaris* spp. 2	-	-	-	12,9	0,65
Asteraceae	*Soliago chilensis*	0,5	5,98	0,06	0,25	-
Fabaceae	*Trifolium pratense*	22,5	-	-	-	-
Anacardiaceae	*Schinus longifolius*	0,1	-	-	-	-
Rhamnaceae	*Scutia buxifolia*	0,1	-	-	-	-
Apiaceae	*Eryngium* spp.	6,7	-	-	-	-
Paoceae	-	1,3	-	-	-	-
Asteraceae	T. *Eupatorium bunifolium*	0,5	0,18	0,31	0,05	-
Asteraceae	*T. cirsium vulgare*	0,2	-	-	-	-
Scrophulariaceae	-	0,2	-	-	-	-
Fabaceae	*Lotus* spp.	36,1	-	-	-	-
Apiaceae	*Ammi viznaga*	2,9	-	-	-	-
Ch-Am	-	0,1	-	-	-	-
Fabaceae	*Glycine max*	3	-	-	-	-
Fabaceae	*Trifolium rapens*	11,1	-	-	-	-

Unknown	-	2	-	-	-	-
Amaryllidaceae	*Allium cepa*	0,7	-	-	-	-
Arecaceae	-	2,1	-	-	-	-
Asteraceae	*Cichorium intybus*	0,2	-	-	-	-
Lamiaceae	*Salvia* spp.	0,1	-	-	-	-
Fabaceae	*Medicago sativa*	5,1	-	-	-	-
Onagraceae	*Ludwigia peploides*	0,8	-	-	-	-

Supplemental Table S2. Proportion of the aminoacids in the polyfloral pollen patty and in the pollen collected by the bees in the E. grandis foraging area. Essential amino acids are highlighted in grey.

	Proportion of aminoacids	
**Aminoacids**	**Polyfloral pollen patty**	**Pollen available in the *E. grandis* foraging area**
Aspartic acid	1.92	1.43
Glutamic acid	1.79	1.59
Serine	0.99	0.81
Glycine	0.81	0.81
Histidine	0.45	0.4
Taurine	0.03	0.04
Arginine	0.94	1.13
Threonina	0.88	0.69
Alanine	1.07	0.95
Proline	2.33	1.95
Tyrosine	0.65	0.54
Valine	0.94	0.8
Methionine	0.33	0.31
Cystein	0.21	0.19
Isoleucine	0.87	0.68
Leucine	1.37	1.15
Phenylalanine	0.75	0.65
Lysine	1.07	1.08
Tryptophane	0.22	0.13

Supplemental Table S3. Pesticides detected in the polyfloral pollen patty, their concentration, the quantification limit of the technique (LOQ), the LD50 for bees according to the Pesticides Properties DataBase, of the University of Hertfordshire (available at https://sitem.herts.ac.uk/aeru/footprint/es/) and the number of times lower than the LD50 after 10 days of pollen consumption according to Brodschneider and Crailsheim 86. ND: non-detected.

Pesticide	Concentration (mg/kg)	LOQ (mg/kg)	LD_{50} after 48hs of consumption (mg/abeja)	Number of times lower than the LD_{50} after 10 days of pollen consumption
Acetamiprid	ND	0.001	-	-
Atrazine	<LOQ	0.001	>0.1	>2,325,581
Azoxystrobin	0.0063	0.0001	>0.2	>738,280
Boscalid	ND	0.001	-	-
Carbaryl	ND	0.001	-	-
Carbendazym	0.0113	0.0001	>0.05	>102,902
Clothianidin	ND	0.0001	-	-
Cipermetrine	ND	0.1	-	-
Chlorfenvinphos (Z+E)	ND	0.05	-	-
Chlorothalonil	ND	0.1	-	-
Chlorpyrifos-ethyl	ND	0.05	-	-
Chlorpyrifos-methyl	ND	0.05	-	-
Coumaphos	0.035	0.0001	0.312	208,000
Dizinon	ND	0.05	-	-
Dimethoate	ND	0.001	-	-
Endosulfan sulfate	ND	0.005	-	-
Fipronil	ND	0.05	-	-
Haloxyfop methyl	ND	0.001	-	-
Hexythiazox	ND	0.001	-	-
Imazalil	ND	0.001	-	-
Imidacloprid	ND	0.001	-	-
Iprodine	ND	0.001	-	-
Lambda cyhalothrin	ND	0.05	-	-
Metornil	ND	0.001	-	-
Metidation	ND	0.1	-	-
p,p-DDE	ND	0.05	-	-
p,p-DDT	ND	0.05	-	-
Pyraclostrobin	<LOQ	0.001	>0.1	>2,325,581
Tau-Fluvalinate	ND	0.05	-	-
Tebuconazole	0.005	0.001	>0.2	>930,233
Thiacloprid	ND	0.001	-	-
Thiamethoxam	ND	0.001	-	-
Trifluralin	ND	0.1	-	-

Supplemental Figure S4. Location of the experimental apiary within the *E. grandis* plantation.

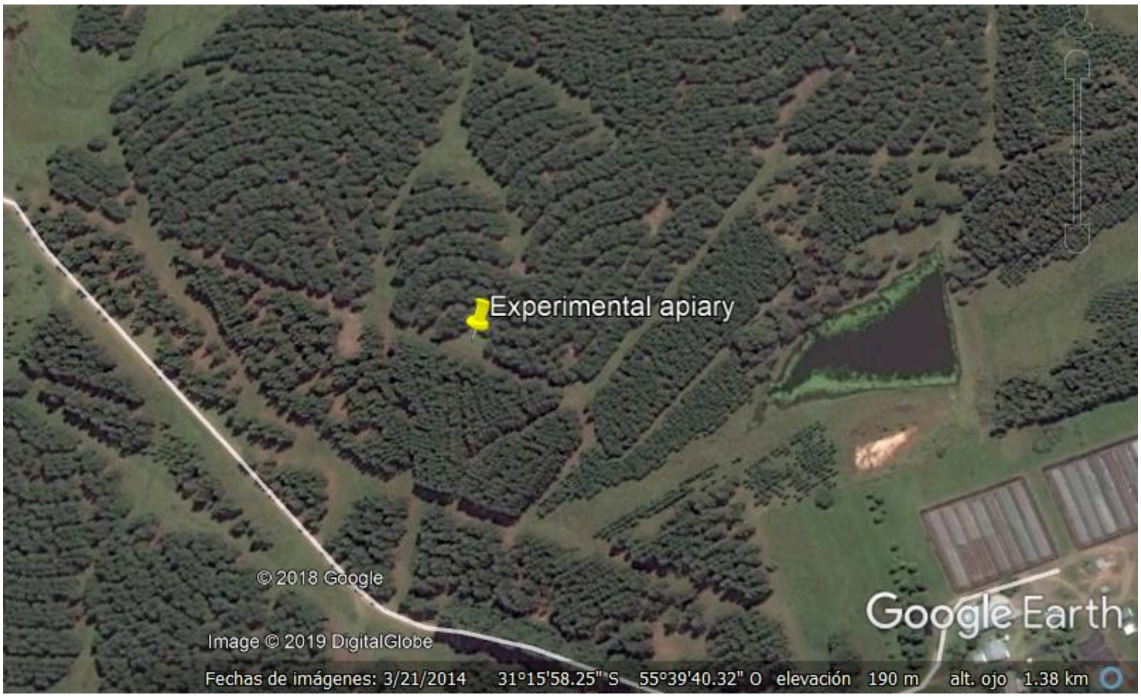

Supplement: Supplementary file 1 — Supplemental material [file 41598_2019_46453_MOESM1_ESM.pdf]
